# Advanced Molecular Imaging (MRI/MRS/^1^H NMR) for Metabolic Information in Young Adults with Health Risk Obesity

**DOI:** 10.3390/life11101035

**Published:** 2021-10-01

**Authors:** Khin Thandar Htun, Jie Pan, Duanghathai Pasanta, Montree Tungjai, Chatchanok Udomtanakunchai, Thanaporn Petcharoen, Nattacha Chamta, Supak Kosicharoen, Kiattisak Chukua, Christopher Lai, Suchart Kothan

**Affiliations:** 1 Center of Radiation Research and Medical Imaging, Department of Radiologic Technology, Faculty of Associated Medical Sciences, Chiang Mai University, Chiang Mai 50200, Thailand; khinthandar_htun@cmu.ac.th (K.T.H.); duanghathai.pas@cmu.ac.th (D.P.); montree.t@cmu.ac.th (M.T.); chatchanok.u@cmu.ac.th (C.U.); thanaporn_petcharoen@cmu.ac.th (T.P.); nattacha_chamta@cmu.ac.th (N.C.); supak_k@cmu.ac.th (S.K.); Kiattisak_chukua@cmu.ac.th (K.C.); 2 Shandong Provincial Key Laboratory of Animal Resistant Biology, College of Life Sciences, Shandong Normal University, Jinan 250014, China; 3 Health and Social Sciences, Singapore Institute of Technology, 10 Dover Drive, Singapore 138683, Singapore; chris.lai@singaporetech.edu.sg

**Keywords:** MRI, MRS, ^1^H NMR, obesity, young adults, metabolic syndrome

## Abstract

Background: Obesity or being overweight is a medical condition of abnormal body fat accumulation which is associated with a higher risk of developing metabolic syndrome. The distinct body fat depots on specific parts of the anatomy have unique metabolic properties and different types of regional excessive fat distribution can be a disease hazard. The aim of this study was to identify the metabolome and molecular imaging phenotypes among a young adult population. Methods: The amount and distribution of fat and lipid metabolites profile in the abdomen, liver, and calf muscles of 46 normal weight, 17 overweight, and 13 obese participants were acquired using MRI and MR spectroscopy (MRS), respectively. The serum metabolic profile was obtained using proton NMR spectroscopy. NMR spectra were integrated into seven integration regions, which reflect relative metabolites. Results: A significant metabolic disorder symptom appeared in the overweight and obese group, and increased lipid deposition occurred in the abdomen, hepatocytes, and muscles that were statistically significant. Overall, the visceral fat depots had a marked influence on dyslipidemia biomarkers, blood triglyceride (r = 0.592, *p* < 0.001), and high-density lipoprotein cholesterol (r = −0.484, *p* < 0.001). Intrahepatocellular lipid was associated with diabetes predictors for hemoglobin (HbA1c%; r = 0.379, *p* < 0.001) and for fasting blood sugar (r = 0.333, *p* < 0.05). The lipid signals in serum triglyceride and glucose signals gave similar correspondence to biochemical lipid profiles. Conclusions: This study proves the association between alteration in metabolome in young adults, which is the key population for early prevention of obesity and metabolic syndrome. This study suggests that dyslipidemia prevalence is influenced mainly by the visceral fat depot, and liver fat depot is a key determinant for glucose metabolism and hyperglycemia. Moreover, noninvasive advanced molecular imaging completely elucidated the impact of fat distribution on the anthropometric and laboratory parameters, especially indices of the metabolic syndrome biomarkers in young adults.

## 1. Introduction

The increasing rates of global obesity have been substantial in the young adult population. About 4.8–7.0% of total metabolic syndrome (MetS) subjects are between 18–30 years of age based on data from the World Health Organization (WHO) statistics [1,2,3]. A medical condition of abnormal accumulation of body fat poses a major risk for obesity-associated problems, cardiovascular disease (CVD), and type 2 diabetes [4]. The high fatty acid transportation rate to non-adipose tissue increases the ectopic fat accumulation that impairs organ function, disturbs glucose and lipid metabolism, which is the pathogenesis of the metabolic syndrome. Due to the inhomogeneous obesity situation, regional fat distribution seems to be a significant indicator of metabolic disease [5,6].

The distinct body fat depots on individual anatomy have unique metabolic properties and different regional excessive fat accumulation leads to a diversity of disease risks [7]. The abdominal adiposity, especially in intraabdominal fat stores shows more pathogenic metabolic syndrome components and it strongly increases statistically higher mortality rates and disease risk disorders [8].

The high visceral fat induces hepatic lipogenesis which has an adverse effect on glucose homeostasis and insulin resistance. Moreover, the redirected flow of free fatty acid (FFA) discharged from visceral lipolysis to the liver in obese people promotes hepatic triglyceride storage, which is the main cause of organ-specific disease nonalcoholic fatty liver disease (NAFLD) [9,10,11]. The fat deposition in the liver is compensated by more energy storage in peripheral adipose tissue according to the “ectopic fat hypothesis” [12]. Ectopic lipids are leading to organ-specific insulin resistance via a process of lipotoxicity [13]. Individuals prone to T2DM show a greater propensity to accumulate visceral white adipose tissue for a given weight, resulting from impaired subcutaneous fat storage capacity. Consequently, with even modest weight gain, they accumulate lipids in visceral and ectopic tissues, such as the liver, leading to marked insulin resistance [14]. Moreover, some individuals, particularly women, despite attaining high BMIs (as high as 50 to 60 kg/m^2^), remain insulin-sensitive, normoglycemic, and normolipidemic. Imaging studies show these individuals have low visceral and ectopic lipids but a high subcutaneous white adipose tissue content [15,16]. The intercorrelations of different regional fat content can reveal the fundamental physiological link in obesity.

Anthropometric assessments (body mass index (BMI), waist circumference (WC), hip circumference (HC), waist-hip ratio (WHR), and abdominal sagittal plane) are used routinely to screen overweight or obesity subjects, although these are not for accurate diagnosis [8,17]. Moreover, adipose tissue quantification and distinguishing lipid metabolites individually in obesity cannot be dealt with using these traditional methods [18]. Therefore, to determine whether the subject has excess fat and an abnormal metabolites condition, further assessment methods need to be established. The accuracy and non-invasiveness of the assessment method are crucial for metabolic profile determination in the modern world. So far, there is no noninvasive gold standard method for body composition and metabolic assessment for obesity.

Computed Tomography (CT) and Magnetic Resonance Imaging (MRI) are modern imaging techniques that have unique capabilities in the noninvasive study of body composition, especially in the quantification of adipose tissue, fat in skeletal muscle, visceral organs, and the brain [19,20]. However, since CT is significantly lower in the accuracy of determining liver fat being less than 5%, this limits its diagnostic possibilities for low-grade steatosis. [21]. The ionizing radiation dose in CT is significant in ethical considerations of research studies on human subjects, and it is not appropriate for longitudinal follow-up studies [22].

MRI and MRS have higher sensitivity and specificity in detecting low cut-off amounts of liver steatosis than CT whenever liver biopsy is used, being an ideal measurement [23]. A complete ectopic fat estimation is done by the total breakdown of net MR signals into fat and water separately in MRI. Moreover, MR results show a very high agreement concordance with CT in abdominal fat estimation [24]. The chemical shift-based MRS shows several metabolites from a single test. It is non-destructive, noninvasive, and its non-targeted characters help to study comprehensive metabolic profiling for a broader understanding of metabolic disorders related to obesity [25]. Metabolomics provides a useful systemic approach to investigate variation in metabolites, response to alterations in genetics, nutrition, environments, and gut microbiota in both humans and animals. These changes in metabolome reflect the changes in cellular activity and allow the prediction of disease development and progression [26].

A non-targeted vitro proton nuclear magnetic resonance (^1^H NMR) has been used for metabolomics study on metabolic syndromes, liver diseases, and metabolite profile studies in diabetes patients. The serum metabonomic could be used to develop biomarkers to identify early obesity and other associated health risks to facilitate the prevention and treatment of obesity [27,28,29]. The choice of proper analytic metabolomics technique is important for the systemic determination of metabolite profiles to gain more understanding of the biochemical changes in obesity or related diseases both in individual organs and at the organism level [30]. Due to the unique character of ^1^H NMR spectroscopy, its high reliability (coefficient of variation ~1–2%), excellent stability, high integration accuracy, and the simultaneous detection of a broad range of metabolites from multicomponent mixtures makes it a feasible technique for metabolomic studies [31,32]. However, this metabolomic approach has not yet been widely applied in clinical practice.

The aim of the study was to establish an ideal assessment and noninvasive diagnostic molecular imaging (MRI/MRS/^1^H NMR) approach to better determine obesity characterization and related abnormal metabolites conditions in the body using young adult human subjects. It may be possible to determine previously unknown metabolic information caused by or related to obesity. A non-targeted NMR-based metabolic profiling in vitro approach was applied to identify and quantify normal weight (NW), overweight (OW), and obese (OB) young adult serum metabolites in this research. Furthermore, as an in vivo study, MR imaging and MR spectroscopy approaches were applied to determine the body lipid composition in the young population.

## 2. Materials and Methods

### 2.1. Study Population

This prospective, cross-sectional study included 46 NW, 17 OW, and 13 OB (29 male and 47 female) volunteers aged 21.5 ± 1.6 years who were chosen from a population residing in Chiang Mai, Thailand. Weight status was based on WHO guidelines. NW has a BMI of 18.5–24.9 kg/m^2^, OW ≥ 25–29.9 kg/m^2^, and OB greater than or equal to 30 kg/m^2^ [33]. The subjects having chronic liver disease, regular medication usage, alcohol consumption > 150g/week, hyperglycemia (fasting blood sugar, FBS > 140 mg/dL), hypertriglyceridemia (triglyceride, TG > 300 mg/dL), athletes, and contraindication for MR were excluded from the study using a questionnaire regarding health, lifestyle status, personal, and family medical history.

### 2.2. Ethical Considerations

The study was conducted following the Ethics Committee of the Faculty of Associated Medical Sciences, Chiang Mai University, Chiang Mai, Thailand (AMSEC-62EX-024). Informed consent was obtained from all participants.

### 2.3. Biophysical Characteristics

The same observer measured every subject wearing examination clothing. Height (cm) and body weight (kg) were measured to the nearest 0.5 cm and 0.1 kg, respectively. WC (cm) and HC (cm) were measured with non-elastic tape while the subject softly exhaled. WC was measured at the midpoint of the distal border of the lower rib and the upper margin of the iliac crest (WHO guideline). HC was measured at the widest section of the buttocks. WHR was calculated from WC divided by HC.

### 2.4. Conventional Biochemical Assay

Blood collection was done by Associated Medical Science Clinical Service Center, Chiang Mai University. Ten milliliters of intravenous blood were drawn from antecubital veins on MR scanning day and was biochemically analyzed using a fully automated analyzer (Architect ci8200, Abbott Diagnostic). The test focused on total cholesterol (TC), low-density lipoprotein cholesterol (LDL-C), high-density lipoprotein cholesterol (HDL-C), TG, FBS, glycated blood glucose (HbA1c), and alanine transaminase (ALT). Subjects were informed about fasting for 10–12 h before blood examination. Later, the LDL-C concentration was calculated from novel adjustable LDL estimation equations [34,35].

The National Cholesterol Education Program (NCEP) Adult Treatment Panel (ATP) III [36] has defined dyslipidemia as TC ≥ 200 mg/dL, TG ≥ 150 mg/dL, LDL-C ≥ 130 mg/dL, and HDL-C ≤ 40 mg/dL. Normal FBS ranges should be between 70–100 mg/dL, and normal HbA1c levels should be less than 6% (2018). The current upper limit of serum ALT, though varied among laboratories, is generally around 40 IU/L [37].

### 2.5. Abdominal and Calf Muscular Fat Composition Determined by MRI

All volunteers were scanned for abdominal MRI by 1.5 Tesla, Ingenia, Philips MR machine (Philips Healthcare, Amsterdam, Netherlands) under comfortable conditions. The T1 weighted turbo spin-echo (TSE) axial images of the abdomen (5 mm × 5 mm × 5 mm) were collected with a sense cardiac coil. An axial slice at L3–4 level was removed from the liver or buttock adipose tissue and was saved using digital imaging and communications in medicine (DICOM) format for fat quantification [38,39]. A 3 mm slice thickness axial image at the lower border of the patella was used for the calf muscle.

**Figure 1:**
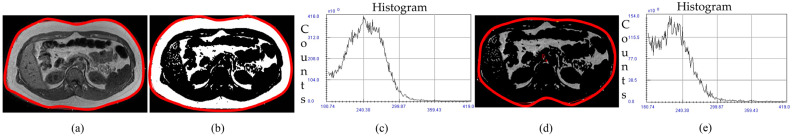
The image was analyzed using the Medical Image Processing, Analysis, and Visualization software; (**a**) T1 weighted DICOM MRI image at L3–L4 level. (**b**) Segmented image for total abdominal adipose tissue and (**c**) related histogram. (**d**) Segmented image for visceral adipose tissue and (**e**) related histogram. The pixel value that appears as white and black in the binary image represents adipose tissue content and soft tissue, respectively. The visceral area was determined through manual drawing in the region of interest. Red line: Region of interest.

The resultant transverse abdominal and leg images were analyzed using the Medical Image Processing, Analysis, and Visualization (MIPAV, National Institutes of Health, Bethesda, MA, USA) software package with a semiautomatic segmentation technique that converts grayscale pixels into binary images of black and white, based on the signal intensity-based histogram-thresholding method [40,41]. The histogram typically shows two peaks of gray values relating to adipose tissue and non-adipose tissue [42]. The area with high signal intensity, or which appears brighter represents adipose tissue, was set as the threshold to exclude the non-relevant organs and tissue in the image, as shown in Figure 1 [43]. The pixel value that appeared as white in the binary image represents adipose tissue content, while the black pixel represents soft tissue such as muscle, blood vessels, and bony structures. The visceral area was determined through the manual drawing in the region of interest (ROI) and the abdominal wall separation between intra-abdominal and extra-abdominal boundaries [44]. After that, visceral fat percentage (Vis fat %) was calculated in total abdominal composition. The fat occupied outside the muscles surrounding the abdominal cavity is subcutaneous adipose tissue. This combination of manual and automated segmentation methods quantified the different abdominal fat distributions. Abdominal subcutaneous fat percentage (Sub fat %) was also calculated by subtracting Vis fat % from total abdominal fat percentage (Abd fat %) [24].

**Figure 2:**
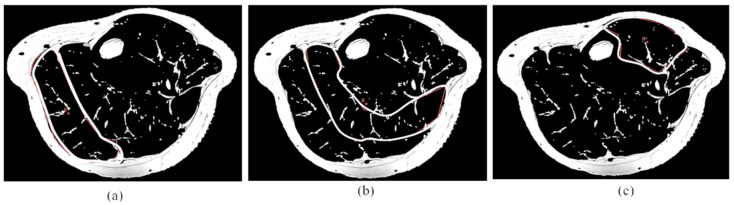
Muscular images were analyzed using the Medical Image Processing, Analysis, and Visualization software; Segmented axial image of (**a**) gastrocnemius, (**b**) soleus, and (**c**) tibialis anterior muscle. The pixel value that appears as white and black in the binary image represents adipose tissue content and soft tissue, respectively. The gastrocnemius, soleus, and tibialis anterior muscle area were determined through manual drawing in the region of interest. White line: Region of interest.

Manual tracings on intermuscular septa between gastrocnemius (G), soleus (S), and tibialis anterior (TA) muscles were done for specific muscle localization as shown in Figure 2. Intensity threshold setting was made with the aid of a histogram and binary grayscale image for fat content calculation of specific muscle, gastrocnemius fat percentage (G-fat %), soleus (S-fat %), and tibialis anterior (TA-fat %). The same image analysis method as abdominal fat detection was used.

### 2.6. Intrahepatocellular and Muscular Lipid Determination by MR Spectroscopy

**Figure 3:**
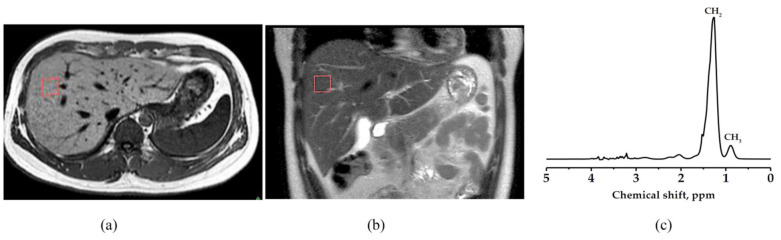
MR spectra were acquired; Liver image with single-voxel (10 mm × 10 mm × 10 mm) (**a**) axial image and (**b**) coronal image. (**c**) MRS spectrum was obtained from the signal in a single voxel with a PRESS sequence. Methyl (CH_3_) peak at 0.9 ppm, Methylene (CH_2_) peak at 1.3 ppm. Red square: Voxe.

MR spectra were acquired by 1.5 Tesla, Ingenia, Philip MR machine (Philips Healthcare, Amsterdam, Netherlands), using a cardiac coil for liver and dedicated knee coil for the left calf muscle. Voxel 10 mm × 10 mm × 10 mm size was carefully positioned on the right lobe of the liver (Couinaud lobe segment V-VIII), avoiding bile ducts and large vascular structures on a 10 mm thick axial image for the liver MRS data acquisition. The spectra were obtained with a single voxel PRESS sequence (TR = 2000 ms, TE = 31 ms, and 128 number of signal averaging) as shown in Figure 3. Intrahepatocellular lipid (IHL) was analyzed by an Origin Pro 2015 (64 bits) Beta3 b9.2.196 analyzer that expressed the peak area.

**Figure 4:**
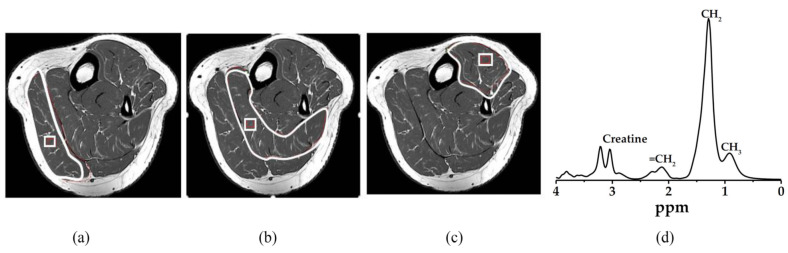
MR spectra were acquired; Voxel (10 mm × 10 mm × 10 mm) localization on (**a**) gastrocnemius muscle, (**b**) soleus muscle, (**c**) tibialis anterior muscle, and (**d**) MRS spectrum with major lipid spectrum peaks CH_3_ (0.9 ppm), CH_2_ (1.3 ppm), and (2.1 ppm). The pixel value that appears as white and black in the binary image represents adipose tissue content and soft tissue, respectively. The gastrocnemius, soleus, and tibialis anterior muscle area were determined through manual drawing in the region of interest. White line: Region of interest, White square: Voxel, CH_3_: Methyl, CH_2_: Methylene.

The same scanning parameters were used for muscular metabolite signals. Voxel 10 mm × 10 mm × 10 mm size was located on gastrocnemius, soleus, and tibialis anterior muscles respectively, avoiding the involvement of the intermuscular lipid and vascular structures as shown in Figure 4. The total triglyceride lipid from methyl (TG-CH_3_) and methylene (TG-CH_2_) protons of the gastrocnemius (G-TG), soleus (S-TG), and tibialis anterior (TA-TG) were obtained using the same method as the IHL analysis. The lipid content differences in each muscle spectrum involved the different fiber type composition and their metabolic activity. Spectrum fitting and quantification were done for water peak (4.7 ppm) and major lipid spectrum peaks -CH_3_ (0.9 ppm), -CH_2_ (1.3 ppm), and (2.1 ppm) with prior knowledge [45].

### 2.7. Sample Collection, Sample Preparation, and ^1^H NMR Spectroscopy

¹H NMR technique was used to measure serum metabolites of NW, OW, and OB groups. Fresh serum samples were removed from −80°C storage and thawed at room temperature. An optimized method was adopted for preparing the samples after centrifuging at 16,000× *g* for 10 min at 4 °C. A 100 μL of supernatant was dissolved by adding 500 μL of dimethyl sulfoxide-D6 with 99.8% deuteration that was mixed gently. The 550 μL of the homogeneous solution was pipetted into 5 mm capillary tubes for NMR measurement. The proton spectrum was acquired at 27 °C on a Bruker AVANCE 500 MHz (Bruker, Germany) with Carr-Purcell-Meiboom-Gill (CPMG, –RD–90°–(t–180°–t) n–acquire) using a water-suppression pre-saturation pulse sequence. A 90° pulse with a 16 number of signal averaging (NSA) was applied. The baseline and phase correction were carefully adjusted by TopSpin 4.0.7 software. Spectra in the 0 to 8 ppm range were analyzed. The spectral data were normalized to the total integrated area prior to the data analysis. The metabolite resonances were assigned based on comparison with existing literature and human databases [46].

### 2.8. Statistical Analysis

After the Kolmogorov-Smirnov test and Shapiro-Wilk Test assessment, One-way ANOVA statistic was performed as univariate analysis to determine the variance in the groups according to biophysical profiles, biochemical results, lipid component detected by MRI/MRS, and ¹H NMR. Value is mean ± SD unless stated otherwise and *p* < 0.05 is considered statistically significant. The relationship of dependent variables (different fat distribution from MRI, MRS) and anthropometric, and blood biochemical parameters were tested by Pearson’s correlation analysis, and the correlation coefficient (r) value and *p*-value were determined. Cohen’s d was calculated as the effect size (between groups). Multiple regression statistical analysis was performed using IBM SPSS version 17 and OriginPro 2015.

## 3. Results

### 3.1. Biophysical and Biochemical Characteristics

The descriptive anthropometric and biochemical characteristics of the study population are presented according to weight status. Overall, OB subjects were identified as being significantly greater in all anthropometric parameters, BMI, WC, HC, and WHR (with *p* < 0.001 in all, and high effect size between groups) than both NW and OW groups. All blood biochemical profiles measured in OB were significantly higher than NW young adults. Only TG, HDL-C, and FBS levels in OW were significantly different from NW as shown in Table 1. No significant differences in blood results between OW and OB except ALT value were found.

### 3.2. Abdominal and Calf Muscular Fat Content Characteristics in NW, OW and OB Young Adults Detected by MRI

Overall, OB young adults have significantly higher Abd fat %, Vis fat %, and S-Fat % when compared to both NW and OW. There was no significant difference in Sub fat %, G-fat %, and TA-fat % between OW and OB young adults. The OW had about 1.8 times higher abdominal fat compartments than NW. The percentage of visceral fat was the greatest difference between OW and OB groups, accounting for 1.89 times, and effect size (Cohen’s d) = 1.70), as shown in Table 2.

The general correlation analysis for all anthropometric variables, biochemical parameters, and ectopic fat storage in various parts of anatomy (abdomen and leg muscle regions) are summarized in Table 3. It shows a strong correlation of Abd fat, Vis fat, and Sub fat with BMI, WC, and HC at a significant level (all *p* < 0.001). BMI, WC, and HC are variables that correlate to the greatest degree with Abd fat % (r = 0.770, r = 0.754, and r = 0.770) among all abdominal fats. For visceral fat, BMI shows the strongest correlated parameter (r = 0.723). HC shows greatest correlation with subcutaneous lipid (r = 0.720). WHR correlates most strongly with Vis fat % (r = 0.588) compared to Abd fat % (r = 0.517) and Sub fat % (r = 0.429). Moreover, the visceral fat depot shows stronger correlation to blood TG (r = 0.592, *p* < 0.001), ALT (r = 0.472, *p* < 0.001) and HDL-C (r = -0.484, *p* < 0.001) than to abdominal and subcutaneous fat compartment. HbA1c moderately correlates to Vis fat % (r = 0.308, *p* < 0.05) and has a slight association strength with Abd fat % (r = 0.255, *p* < 0.05) but no relation to Sub fat %. FBS has no relation to all abdominal fat depots at all.

The S-fat has the strongest significant association with biometric measurements, BMI, WC, HC, and WHR (r = 0.666, r = 0.619, r = 0.639, r = 0.408, all *p* < 0.001) compared to both G-fat and TA-fat as shown in Table 3. Blood TG, ALT and HDL-C levels are moderately correlated to S-fat (r = 0.370, r = 0.418, r = −0.415, *p* < 0.05 and *p* < 0.001), but G-fat and TA-fat have a lower relationship. Only S-fat is associated with the blood total cholesterol (r = 0.240, *p* < 0.05). According to correlation coefficient “r” values, the anthropometric parameters association with all compartmental fat deposited in the abdomen are greater than that of fat accumulation in the leg muscles.

### 3.3. Intrahepatocellular and Intramuscular Lipid Content Characteristics in NW, OW and OB Young Adults Detected by MRS

**Figure 5:**
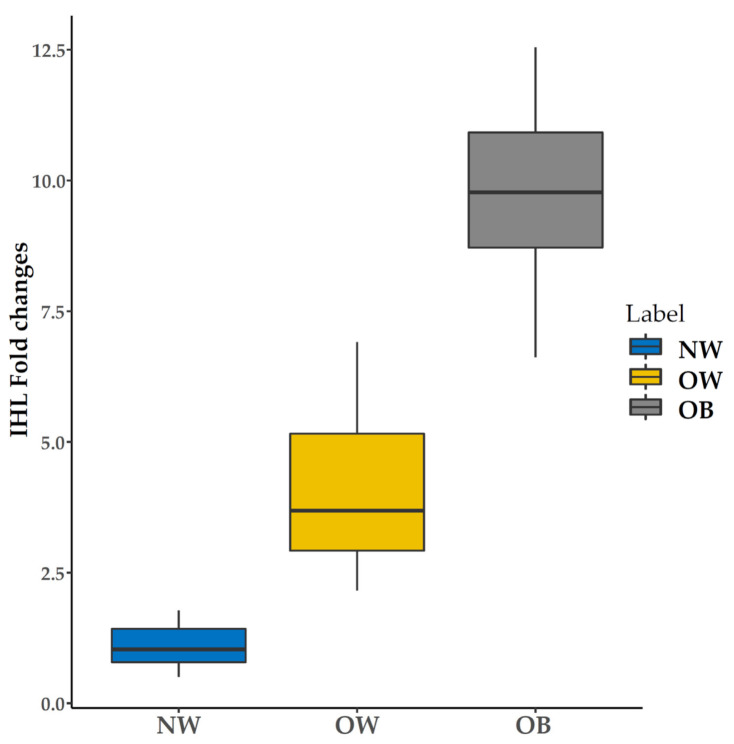
Intra-hepatocellular lipid content characteristics between NW (normal weight), OW (overweight), and OB (obese) groups.

The IHL content in OB and OW is significantly higher than NW. After normalization, IHL in OB is almost 9.4 fold and in OW reaches about 4.1 fold greater than NW, respectively, as shown in Figure 5. In this study, the metabolic evidence between males was increasing different from females, particularly HDL-C, LDL-C, ALT, but no difference in HbA1c %. For lipid levels in MR images, Vis fat % and MRS-derived liver fat were higher in males than females (data not shown).

**Figure 6:**
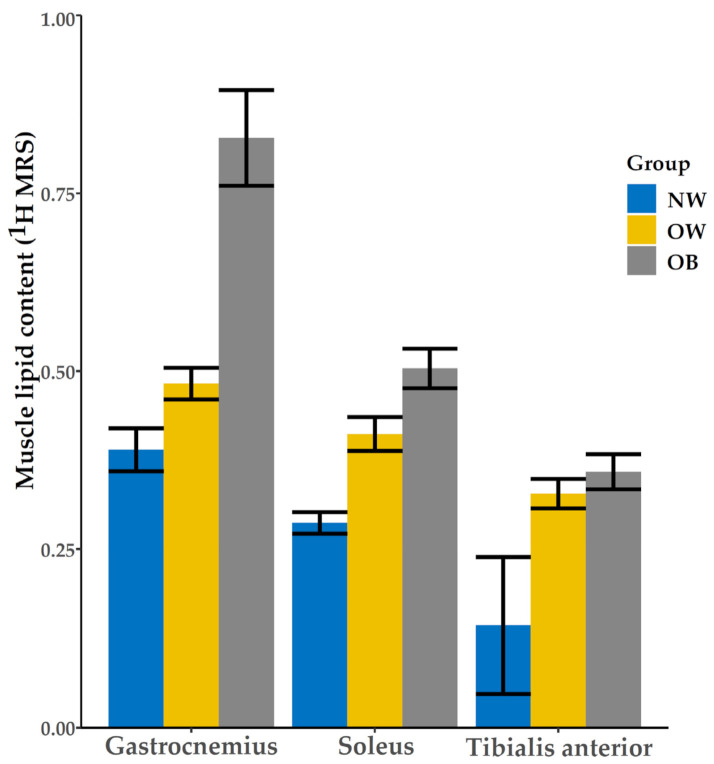
Muscular lipid content characteristics between NW (normal weight), OW (overweight), and OB (obese) groups in gastrocnemius, soleus, and tibialis anterior muscles.

The amount of intra-myocellular lipid accumulation in each calf muscle of the three groups is different. In the gastrocnemius, there is a significant difference between OB and OW. The other two muscles also showed significant differences between NW and OW and OB individually as shown in Figure 6.

**Figure 7:**
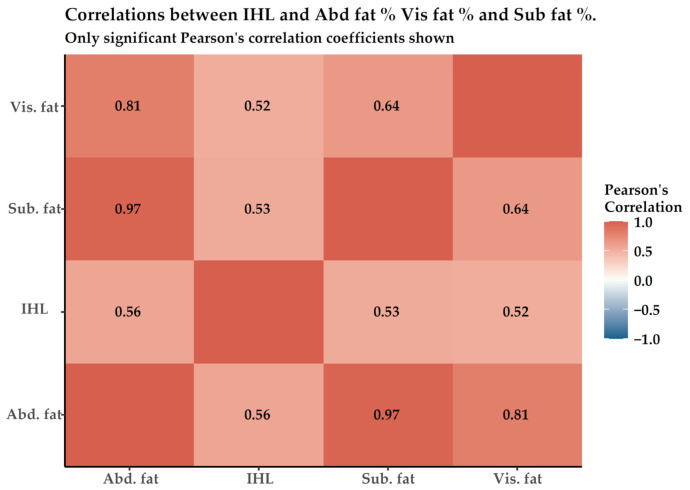
Linear correlations between IHL, Abd fat % Vis fat %, and Sub fat %. All data are presented as “r” values of Pearson correlation. Abd fat, Abdominal fat; Vis fat, visceral adipose tissue; Sub fat, Subcutaneous fat; IHL, intrahepatocellular lipid.

There were high degrees of associations between IHL content and BMI (r = 0.658), WC (r = 0.675), HC (r = 0.583), and WHR (r = 0.610); all *p* < 0.001. IHL showed a positive correlation with both HbA1c (r = 0.379, *p* < 0.001) and FBS (r = 0.333, *p* < 0.05) levels and negative association with HDL-C (r = −0.403, *p* < 0.001). The liver function indicator, represented as blood ALT level is correlated moderately with IHL (r = 0.480, *p* < 0.001), whereas TG is weakly related to IHL (r = 0.357, *p* < 0.05). With regard to association of IHL with abdominal ectopic fat stores, IHL demonstrated strong associations with all abdominal lipid stores, Abd fat %, Vis fat %, and Sub fat % (r = 0.563, 0.518, 0.529 and all *p* < 0.001) as shown in Figure 7.

The TG contents in all calf muscles, gastrocnemius, soleus, and tibialis anterior are correlated with BMI, WC, and HC. G-TG had the strongest correlation when compared to the other two, as shown in Table 4. WHR was greatly associated with S-TG (r = 0.425, *p* < 0.001) more so than G-TG (r = 0.367, *p* < 0.05) and TA-TG (r = 0.374, *p* < 0.001). Moreover, S-TG alone was associated with HbA1c (r = 0.305, *p* < 0.05). There were moderate to weak correlations of G-TG, S-TG, and TA-TG with TG, ALT, and negative relation parameters with HDL-C, as seen in Table 4.

### 3.4. ^1^H NMR Serum Lipid Metabolite Characteristics in NW, OW, and OB Young Adults

**Figure 8:**
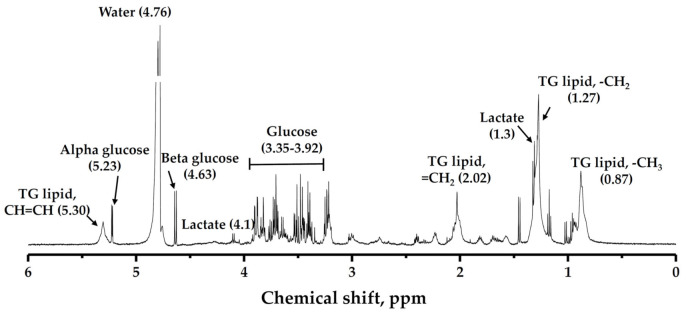
^1^H NMR serum spectrum that indicates the proton peaks of lipid and glucose metabolites.

A zoom-in of a typical ^1^H NMR spectrum of blood serum is illustrated in Figure 8, showing the different chemical shifts (ppm) related to the proton signal of triglyceride lipid and glucose metabolites. The lipid and glucose metabolite differences, the changing trend between NW, OW, and OB young adults are presented in Table 5.

Pearson’s correlation of different ectopic fats detected by MRI/MRS were significant and strong associations with ^1^H NMR lipid biomarkers were noted. Among them, the visceral fat compartment had the highest degree of association with all TG lipid variables. Additionally, all abdominal fat compartments had mild relation to only beta-glucose, one of the glycolysis biomarkers. In ^1^H NMR results, only unsaturated TG lipid (=CH_2_) was moderately associated with calf muscle fat content. The IHL was significantly associated with all TG metabolite variables and glucose variables. The glucose variables had the greatest correlation coefficients compared to lipid variables. The correlation coefficient values are shown in Table 6 and Table 7.

### 3.5. Multiple Regression Analyses

A multiple regression analysis was done to estimate the relationship between quantitative lipid contents collected from MRI/MRS (set as dependent variables) and more than one biophysical and biochemical parameter (set as independent variables) using a straight line after age adjustment. In this regression model, R^2^ shows the proportion of the variance for individual dependent variables explained by different independent variables. The higher R^2^ value represents smaller differences between the observed data and the fitted values. According to results of multiple regression, as shown in Table 8, BMI stands out as the best predictor for overall abdominal adiposity and subcutaneous adipose tissue depot while WC contributed the most to visceral and intrahepatic lipid. Plasma TG was the significant determinant for high metabolic active intra-abdominal adipose tissue. Despite different degrees of correlations of independent anthropometric variables with different skeletal muscle types, only soleus adipose tissue has the highest determinant power with a BMI of about 44.5% (not expressed in the result tables).

## 4. Discussion

There is no doubt that the total body adiposity and different compartmental fat distribution are the main factors for metabolic disease development. Particularly, central obesity is a major role in insulin resistance, diabetes, dyslipidemia, inflammation, hypertension, and CVD [1,47]. The storage of surplus triglyceride in non-adipose tissue is the major consideration to determine metabolic alteration [48]. The investigation and monitoring of metabolic biomarkers and early detection of metabolic information related to obesity are of great importance to halt further progression of the diseases and for initiating any anti-obesity treatment plans [49].

Anthropometric parameters, BMI, skinfold thickness, or WC are physical assessments that have been used routinely in previous epidemiologic studies done on obesity and metabolic syndrome screening [50]. In this study, almost all anthropometric variables correlated in different degrees with white fat distribution in the abdomen, liver, and skeletal muscles. Both BMI and WC differences between NW and OB were the highest. Moreover, BMI is strongly related to both total abdominal fat and visceral fat depositions. In this study, the visceral adiposity is the most remarkable fat depot in the abdominal area compared to the subcutaneous and overall abdominal depot. These findings were consistent with a validation study that reported that BMI correlates with WC, which itself appears to be closely related to visceral adipose tissue deposition and metabolic variables [51,52]. Although there is a consideration about the metabolic syndrome criteria proposed regarding abdominal circumference, CT images at WC can almost precisely show the total volume of visceral fat within the abdominal cavity [53]. This study demonstrated that BMI and WC are criteria that are more likely to predict the metabolic syndrome and to predict total abdominal fat and visceral fat amounts. Allison et al. also suggested that WC should be used together as screening tools in clinics to predict the risk of metabolic syndrome in adults [17]. The results of multiple regression analysis determined that BMI is a more powerful variable for overall abdominal fat, and WC is a good screening predictor for visceral fat depots.

According to the results, IHL was linearly associated mostly with WC. WC measurement had a higher association with IHL than WHR. These controversial results report that BMI is a potential marker for liver fat content in young adults. However, the relationship between BMI and IHL is also affected by racial group and genetic background in specifically related genes [54,55]. This study makes it increasingly clear that WC is a better reflection of intrahepatic fat accumulation confirmed by multiple regression analysis after age adjustment.

Inter-muscular and intramuscular fat accumulation are less likely to be influenced by anthropometric parameters, not just in response to over-eating, but mainly coordinate with muscular fiber type composition and their oxidative activity. Our study shows that the relationship between TG and BMI and WC in muscle is not as strong as that of abdominal and liver fat content correlation. In contrast, Forouhi determined that TG was not significantly correlated with BMI in the Asian population compared to Europeans, even though higher TG values are found in Asia [56]. In addition, Hwang et al. did not find a significant correlation of TG and muscular fat content with BMI, except for soleus muscular adipose tissue content [57]. Consistent with this study, BMI had the strongest relationship parameter (r = 0.666, *p* < 0.001) with S-fat % among other muscle relationships, and multiple regression power yielded the same outcomes in this study.

Dyslipidemia and obesity are the most common complex metabolic disorders, leading to type 2 diabetes mellitus (T2DM) and CVD. An 80% of T2DM relates to metabolic syndrome, characterized by hyperglycemia [58]. Hypertension, elevated serum triglycerides, low serum HDL, and insulin resistance are common clinical indicators to resolve MetS related to obesity. The present study showed that HbA1c % correlated with Vis fat % to a higher degree than that with Abd fat %, but no relationship was apparent with Sub fat %. This clearly demonstrates that the visceral and subcutaneous have different functional metabolic activity on glucose homeostasis. It is supportive evidence of the notion that visceral fat is a major cause in the development of metabolic syndrome, more so than subcutaneous adiposity, and is the main corresponding feature of insulin resistance [8].

Although there is no relation to FBS, visceral fat associations with TG and HDL-C were the strongest among all relations in the study. This means that visceral fat has a marked influence on lipid metabolism. This fact confirmed the highest strength relation of visceral fat in MRI to all TG lipid metabolites from the NMR spectrum study. It is probably due to it being less sensitive to insulin activity that leads to a higher fat degradation rate [59]. The normal plasma FFA level is maintained by balancing between the FFA discharge from lipolysis and TG clearance via lipoprotein lipase activity. The visceral fat lipolysis discharges three times more FFA into portal veins in the obese than in normal subjects [9]. This redirected fatty acid to the liver promotes dyslipidemia, characterized by elevated plasma FFA, TG, and LDL, and the reduction of HDL.

When the liver de nova lipogenesis (DNL) rate is higher than rates of TG transportation in VLDL and lipid oxidation, surplus TG is stored in hepatocytes and becomes liver steatosis. It is associated with adverse alterations in glucose, fatty acid, and lipoprotein mechanisms [60]. Releasing the rate of FFA into circulation is directly proportional to body fat mass. The large plasma FFA in blood originates from visceral fat lipolysis in obese subjects and is 20% greater in obese than in lean types [9]. In addition, gene expression of hepatic lipase and hepatic lipoprotein lipase (LPL) are higher in obese than that found in normal-weight people. This promotes the blood-taking rate of FFA to go up by the liver and contributes to hepatocellular fat accumulation [26,61]. This inter-physiological relationship is proven to be a positive and significant one in cross-correlation analysis between lipid distribution in the liver by MRS and in abdominal regions by MRI as shown in Figure 7.

The liver synthesizes fatty acids through complex cytosolic polymerization and undergoes several cycles of metabolic reactions to form one palmitate molecule. About 1–2 g/d of VLDL-TG are incorporated with 5% of fatty acid that is secreted in normal subjects from liver DNL. However, the VLDL-TG secretion rate in obese subjects is much higher, and more fatty acids account for 15–23%. Moreover, Fabbrini et al. demonstrated that the rate of VLDL-TG secretion was twice as great in non-diabetic obese subjects with NAFLD than in those with normal IHL levels [62]. Our results showed that IHL was directly associated with both plasma TG and HDL-C and all TG lipid in the NMR study, as well. Therefore, this means that higher liver fat has a greater impact on lipid homeostasis.

The oxidative stress existing in liver fat infiltration in obese young adults is always accompanied by attenuation of islet β-cell function [63]. Hence, fat in the liver makes that organ less responsive to insulin and leaves too much glucose in the blood, leading to T2DM [64]. It appears that triglyceride accumulation in the liver contributes to hepatic insulin resistance, and individuals with hepatic steatosis subsequently have further development to diabetes [65]. In this study, neither detected regional adipose tissue related to cross-sectional fasting biochemical blood glucose levels except for IHL. IHL had the highest linear correlation with both HbA1c and FBS. Furthermore, the positive relation of IHL with all glucose tributaries was the strongest among all relations in the NMR study. Due to the strongest association with alpha glucose, beta glucose, and total glucose, this confirmed that IHL dominantly regulates glucose homeostasis and more than lipid metabolism.

HDL-C transports excess cholesterol from extra-hepatic periphery tissue to the liver, reducing cholesterol accumulation and plaque formation in the arteries. Therefore, HDL-C, a biomarker for CVD, plays a critical role in cholesterol homeostasis [66]. Moreover, this depends on the degree and distribution in the body. Good HDL cholesterol concentration is inversely related to the abdominal circumference or central obesity. In this study, HDL-C had a reversal association with all MR lipid content results. Among them, Vis fat % had the highest negative relation. This is consistent with the previous remark that a lower HDL-C level in abdominal and visceral obesity is the most significant compared to thigh fat deposition [67]. Nieves DJ et al. also reported that the larger visceral fat area detected by computed tomography was the main indicator for lower HDL-C concentration [68]. Although the use of NMR cannot differentiate the individual serum cholesterol metabolites, the high magnetic field strength of NMR machines and other sample acquisition techniques may determine the cholesterol metabolites in the serum.

The marked elevation of aminotransferase enzyme ALT is a major precursor for the subsequence phase of nonalcoholic fatty liver disease, hepatic steatohepatitis, and liver diseases. In this study, IHL correlated positively and strongly with biochemical ALT profiles, and visceral fat association followed IHL in terms of strength of correlation. This IHL and liver enzyme association can exclude the impact on the endocrine gland nature of hepatocytes which go further as a consequence of hepatic dysfunction and attenuation of pancreatic β-cell function [63]. All other intervening MRI and MRS results were moderately associated with ALT except for TA-fat %. This meant that the serum ALT levels are significantly correlated with various fat depot indices in healthy adults. It is consistent with previous reports that liver enzyme activity is significantly correlated with the high body fat mass group in adults [69]. Perlemuter et al. reported that ALT was inversely related to leg fat mass, and the MRI and MRS leg muscular lipid associations were not as strong as central fat accumulation [70]. This is more likely to compensate for obesity-related liver damage. It was confirmed with no or even slight association of TG and glucose NMR metabolites with muscular fat.

In human calf muscles, normally, slow-twitch fibers (soleus) have higher TG content and are more metabolically active than other glycolytic muscles (gastrocnemius and tibialis anterior) in sedentary and diabetic volunteers [71]. In contrast, the S-TG amount was lower than G-TG in the present study. There was no relation of HbA1c with all skeletal muscle fat except only S-TG. Based on earlier studies, even though TG and HDL-C are related to muscular fat, associations were not found to be as strong as fat depots in the abdomen and liver. This is consistent with the notion that the incidence of peripheral obesity in MetS is lower than that of central obesity [72]. This point was supported by the poor association of all studied muscular fat content from both MRI and MRS with only TG lipid (=CH_2_) from NMR results. Therefore, this study reveals that muscular fat is less likely to influence metabolic activity than body fat in young adults. In this study, we selected a sample of Asian (Thai) people. There were some differences between Asian and European and American obesity types in obesity types and dietary habits as well as genetics, but the abnormal accumulation of white fat was similar, and their lipid profiles also tended to be the same.

Overall, this molecular imaging study evaluated abdominal fat components and intrahepatic lipids as the main joint determinant and can be used to identify young adults with increased potential for metabolic risk. In addition, it shows anthropometric parameters that can be used as conventional predictors of fat deposition in different regions. The study concluded that the abnormal accumulation of white fat in the internal organs and abdomen is more related to obesity-related systemic lipid metabolism disorders, and its importance is far greater than the accumulation of fat in peripheral tissues.

## 5. Conclusions

In this study, we determined the content of ectopic lipid metabolites in the abdomen, liver, and skeletal muscles through noninvasive molecular imaging MRI/MRS technology. We also determined that it closely correlates with conventional blood biochemical and metabolomics serum ^1^H NMR data. Our study suggests that elevated liver fat might be treated as a simple biomarker of hyperglycemia and that visceral fat might be a dyslipidemia-treated biomarker, especially in young obese adults. Moreover, noninvasive advanced molecular imaging techniques completely elucidated the impact of fat distribution on the anthropometric and laboratory parameters, especially indices of MetS, dyslipidemia, and hyperglycemia in young adults. The present study provides clinical diagnostic information for predicting potential metabolic risks in obese subjects.

## Figures and Tables

**Table 1:** Descriptive characteristics of biophysical and biochemical profiles between NW, OW, and OB groups.

Parameters	NW	OW	OB	NW vs. OW	NW vs. OB	OW vs. OB
	Mean ± SD	Mean ± SD	Mean ± SD	*p*, Effect SIZE (between Group)		
Age (years)	21.74 ± 0.82	21.76 ± 1.2	21.23 ± 2.09	0.998, (0.02)	0.382, (−0.32)	0.462, (−0.31)
BMI (kg/m^2^)	20.28 ± 2.15	27.48 ± 1.72	34.40 ± 3.59	<0.001, (3.70)	<0.001, (4.76)	<0.001, (2.45)
WC (cm)	73.37 ± 6.9	88.76 ± 5.76	107.08 ± 9.18	<0.001, (2.42)	<0.001, (4.15)	<0.001, (2.39)
HC (cm)	92.35 ± 6.05	106.53 ± 6.29	116.08 9.38	<0.001, (2.30)	<0.001, (3.00)	<0.001, (1.20)
WHR	0.79 ± 0.05	0.83 ± 0.04	0.92 ± 0.05	<0.05, (0.07)	<0.001, (0.23)	<0.001, (1.77)
TC (mg/dL)	196.43 ± 21.3	197.44 ± 23	218.46 ±37.18	0.991, (0.05)	<0.05, (0.73)	0.079, (0.68)
TG (mg/dL)	77.71 ± 27.72	103.71 ± 51.94	130.31 ± 24.97	<0.05, (0.62)	<0.001, (1.99)	0.095, (0.65)
HDL-C (mg/dL)	63.35 ± 12.02	52.71 ± 8.74	47.15 ± 9.81	<0.05, (−1.01)	<0.001, (−1.48)	0.363, (−0.60)
LDL-C (mg/dL)	123.55 ± 23	124.49 ±20.83	145.25 ± 30.71	0.990, (0.04)	<0.05, (0.79)	0.062, (0.79)
ALT (U/L)	12.49 ±4.10	18.88 ± 9.21	38.55 ± 21.33	0.062, (0.90)	<0.001, (1.70)	<0.001, (1.20)
FBS (mg/dL)	84.67 ± 4.18	87.81 ± 4.62	91.46 ± 5.36	<0.05, (0.72)	<0.001, (1.41)	0.083, (0.73)
HbA1c %	5.09 ± 0.23	5.15 ± 0.13	5.28 ± 0.23	0.561, (0.31)	<0.05, (0.79)	0.210, (0.67)

All data are presented as mean and standard deviation values. NW, normal-weight group; OW, overweight and OB, obese group. BMI, body mass index; WC, waist circumference; HC, hip circumference; WHR, waist to hip ratio; HbA1c, glycated blood glucose; TC, total cholesterol; TG, triglyceride; HDL-C, high-density lipoprotein cholesterol; LDL-C, low-density lipoprotein cholesterol; ALT, alanine transaminase; and FBS, fasting blood sugar.

**Table 2:** Abdominal fat content and muscular fat content characteristics between NW, OW, and OB groups.

Fat % Detected by MRI	NW	OW	OB	NW vs. OW	NW vs. OB	OW vs. OB
	Mean ± SD	Mean ± SD	Mean ± SD	*p*, Effect Size (between Group)		
Abd fat %	17.04 ± 8.02	31.56 ± 8.83	46.88 ± 17.43	<0.001, (1.72)	<0.001, (2.20)	<0.001, (1.11)
Vis fat %	4.14 ± 2.33	7.31 ± 3.93	13.83 ± 3.75	<0.05, (0.98)	<0.001, (3.10)	<0.001, (1.70)
Sub fat %	12.36 ± 6.57	23.30 ± 7.17	31.52 ± 14.52	<0.001, (1.59)	<0.001, (1.70)	0.029, (0.72)
G-fat %	3.26 ± 1.56	5.38 ± 2.69	6.06 ± 2.52	<0.05, (0.96)	<0.001, (1.34)	0.64, (0.26)
S-fat %	3.06 ± 1.41	4.72 ± 1.37	6.89 ± 2.35	<0.05, (1.19)	<0.001, (1.98)	<0.05, (1.13)
TA-fat %	3.35 ± 1.94	5.33 ± 2.79	5.89 ± 3.70	<0.05, (0.82)	<0.05, (0.86)	0.821, (0.17)

Calculated using independent two samples *t*-test and presented as mean and SD (standard deviation). Significant values are shown as *p*-value < 0.05 and <0.001. Abd fat, abdominal fat; Vis fat, visceral fat; Sub fat, subcutaneous fat; G fat, gastrocnemius fat; S fat, soleus fat; TA fat, tibialis anterior fat.

**Table 3:** Pearson’s correlation of different ectopic fats in abdomen and calf muscle with biochemical profiles.

Parameters	Abd Fat %	Vis Fat %	Sub Fat %	G-Fat %	S-Fat %	TA-Fat %
	r	r	r	r	r	r
BMI (kg/m^2^)	0.767 **	0.723 **	0.697 **	0.494 **	0.666 **	0.451 **
WC (cm)	0.754 **	0.718 **	0.680 **	0.477 **	0.619 **	0.437 **
HC (cm)	0.770 **	0.668 **	0.720 **	0.498 **	0.639 **	0.524 **
WHR	0.517 **	0.588 **	0.429 **	0.295 *	0.408 **	0.183
TC (mg/dL)	0.055	0.087	0.046	0.103	0.240 *	0.09
TG (mg/dL)	0.445 **	0.592 **	0.340 *	0.247 *	0.370 *	0.246*
HDL-C (mg/dL)	−0.453 **	−0.484 **	−0.386 **	−0.361 *	−0.415 **	−0.272 *
LDL-C (mg/dL)	0.120	0.130	0.109	0.127	0.035	0.025
ALT (U/L)	0.360 *	0.472 **	0.271 *	0.298 *	0.418 **	0.15
FBS (mg/dL)	0.053	0.088	0.03	0.084	0.106	0.077
HbA1c %	0.255 *	0.308 *	0.212	0.118	0.199	0.218

All data are presented as “r” values of Pearson linear correlation. Significant values are shown as * *p* < 0.05, ** *p* < 0.00.

**Table 4:** Pearson’s correlation of intra-hepatocellular and muscular lipids of G, S, and TA muscle with biochemical profiles.

Parameters	IHL	G-TG	S-TG	TA-TG
	r	r	r	r
BMI (kg/m^2^)	0.658 **	0.548 **	0.479 **	0.458 **
WC (cm)	0.675 **	0.579 **	0.485 **	0.449 **
HC (cm)	0.583 **	0.590 **	0.436 **	0.423 **
WHR	0.610 **	0.367 *	0.425 **	0.374 **
TC (mg/dL)	0.050	0.003	0.059	0.107
TG (mg/dL)	0.357 *	0.278 *	0.279 *	0.483 **
HDL-C (mg/dL)	−0.403 **	−0.398 *	−0.349 *	−0.272 *
LDL-C (mg/dL)	0.148	0.035	0.039	0.105
ALT (U/L)	0.480 **	0.276 *	0.386 **	0.313 *
FBS (mg/dL)	0.333 *	0.086	0.032	0.119
HbA1c %	0.379 **	0.052	0.305 *	0.165

All data are presented as “r” values of Pearson linear correlation. Significant values are shown as * *p* < 0.05, ** *p* < 0.001.

**Table 5:** ^1^H NMR serum triglyceride lipid and glucose characteristics between NW (normal weight), OW (overweight), and OB (obese) groups.

^1^H NMR Serum	ppm	NW	OW	OB	Nor vs. OW	Nor vs. OB	OW vs. OB
		Mean ± SD	Mean ± SD	Mean ± SD	*p*	*p*	*p*
TG lipid (CH=CH)	5.30	0.031 ± 0.004	0.032 ± 0.007	0.033± 0003	0.686	0.419	0.903
TG lipid (=CH_2_)	2.02	0.058 ± 0.006	0.064 ± 0.009	0.072 ± 0.006	<0.05	<0.001	<0.05
TG lipid(-CH_2_)n	1.27	0.129 ± 0.023	0.153 ± 0.042	0.169 ± 0.027	<0.05	<0.001	0.366
TG lipid (-CH_3_)	0.87	0.131 ± 0.011	0.134 ± 0.012	0.135 ± 0.010	0.77	0.542	0.92
Total TG lipid	-	0.349 ± 0.037	0.382 ± 0.067	0.410 ± 0.035	<0.05	<0.001	0.294
Alpha glucose	5.23	0.016 ± 0.001	0.017 ± 0.003	0.018 ± 0.002	0.442	<0.05	0.276
Beta glucose	4.63	0.017 ± 0.002	0.017 ± 0.003	0.018 ± 0.002	0.987	0.79	0.898
Total glucose	3.35–3.92	0.332 ± 0.027	0.347 ± 0.052	0.355 ± 0.036	0.597	0.192	0.771

Calculated using independent two samples *t*-test and presented as mean and SD (standard deviation). NW (normal weight), OW (overweight), and OB (obese). Significant values are shown as *p*-value < 0.05 and <0.001.

**Table 6:** Pearson’s correlation of different ectopic fats detected by MRI with ^1^H NMR metabolite profiles.

^1^H NMR Lipid/MRI Lipid	Abd Fat %	Vis Fat %	Sub Fat %	G-Fat %	S-Fat %	TA-Fat %
	r	r	r	r	r	r
TG lipid (CH=CH)	0.440 **	0.605 **	0.442 **	0.128	0.09	0.12
TG lipid (=CH_2_)	0.575 **	0.880 **	0.564 **	0.333 *	0.483 **	0.247 *
TG lipid(-CH_2_)n	0.662 **	0.766 **	0.669 **	0.217	0.184	0.218
TG lipid (-CH_3_)	0.426 **	0.533 **	0.440 **	0.03	0.001	0.024
Total TG lipid	0.653 **	0.757 **	0.662 **	0.197	0.197	0.223
Alpha glucose	0.227	0.201	0.233 *	0.092	0.089	0.007
Beta glucose	0.345 *	0.297 *	0.357 *	0.08	0.038	0.085
Total glucose	0.036	0.007	0.043	0.009	0.044	0.029

All data are presented as “r” values of Pearson linear correlation. Significant values are shown as * *p* < 0.05, ** *p* < 0.001.

**Table 7:** Pearson’s correlation of different lipid content detected by MRS with ^1^H NMR metabolite profiles.

^1^H NMR Lipid/MRS Lipid	IHL	G-TG	S-TG	T-TG
	r	r	r	r
TG lipid (CH=CH)	0.320 *	0.175	0.06	0.078
TG lipid (=CH_2_)	0.597 **	0.303 *	0.360 *	0.387 **
TG lipid(-CH_2_)n	0.541 **	0.099	0.119	0.209
TG lipid (-CH_3_)	0.259 *	0.202	0.151	0.107
Total TG lipid	0.529 **	0.125	0.075	0.149
Alpha glucose	0.831 **	0.135	0.007	0.003
Beta glucose	0.736 **	0.304 *	0.171	0.068
Total glucose	0.790 **	0.113	0.158	0.162

All data are presented as “r” values of Pearson linear correlation. Significant values are shown as * *p* < 0.05, ** *p* < 0.001.

**Table 8:** Multiple regression analysis estimates the relationship between quantitative lipid contents and more than one biophysical and biochemical parameter.

**Abdominal Fat % as Dependent Variable**											
**Model 1**	**Beta (SE)**	**Sig.**	**R^2^**	**Model 2**	**Beta (SE)**	**Sig.**	**R^2^**	**Model 3**	**Beta (SE)**	**Sig.**	**R^2^**
BMI	0.872 (0.292)	0.001 *	0.621	WC	0.815 (0.120)	0.001 *	0.593	HC	0.782 (0.142)	0.001 *	0.603
TG	0.049 (0.035)	0.593		TG	0.114 (0.035)	0.223		TG	0.074 (0.035)	0.396	
HDL-C	0.043 (0.12)	0.656		HDL-C	0.066 (0.127)	0.523		HDL-C	−0.042 (0.126)	0.464	
ALT	−0.219 (0.074)	0.03		ALT	−0.156 (0.074)	0.122		ALT	0.057 (0.069)	0.646	
HbA1c	0.113 (5.117)	0.178		HbA1c	0.052 (5.28)	0.549		HbA1c	0.079 (5.215)	0.505	
**Visceral Fat % as Dependent Variable**											
**Model 1**	**Beta (SE)**	**Sig.**	**R^2^**	**Model 2**	**Beta (SE)**	**Sig.**	**R^2^**	**Model 3**	**Beta (SE)**	**Sig.**	**R^2^**
BMI	0.557 (0.094)	0.001 *	0.591	WC	0.552 (0.037)	0.001 *	0.595	HC	0.429 (0.047)	0.001 *	0.551
TG	0.279 (0.011)	0.005		TG	0.315 (0.011)	0.001 *		TG	0.313 (0.012)	0.002 *	
HDL-C	0.013 (0.039)	0.897		HDL-C	0.040 (0.039)	0.696		HDL-C	0.003 (0.042)	0.980	
ALT	0.011 (0.024)	0.915		ALT	0.039 (0.023)	0.693		ALT	0.142 (0.023)	0.151	
HbA1c	0.080 (1.649)	0.355		HbA1c	0.041 (1.636)	0.634		HbA1c	0.044 (1.721)	0.624	
**Subcutaneous Fat % as Dependent Variable**											
**Model 1**	**Beta (SE)**	**Sig.**	**R^2^**	**Model 2**	**Beta (SE)**	**Sig.**	**R^2^**	**Model 3**	**Beta (SE)**	**Sig.**	**R^2^**
BMI	0.903 (0.238)	0.001 *	0.538	WC	0.824 (0.099)	0.001 *	0.494	HC	0.831 (0.113)	0.001 *	0.536
TG	−0.042 (0.028)	0.676		TG	0.028 (0.029)	0.786		TG	−0.016 (0.028)	0.874	
HDL-C	0.059 (0.098)	0.582		HDL-C	0.074 (0.105)	0.517		HDL-C	0.100 (0.100)	0.362	
ALT	−0.295 (0.060)	0.009 *		ALT	−0.221 (0.061)	0.050		ALT	−0.117 (0.055)	0.242	
HbA1c	0.123 (4.168)	0.184		HbA1c	0.059 (4.347)	0.536		HbA1c	0.064 (4.159)	0.483	
**Intra-Hepatocellular Lipid as Dependent Variable**											
**Model 1**	**Beta (SE)**	**Sig.**	**R^2^**	**Model 2**	**Beta (SE)**	**Sig.**	**R^2^**	**Model 3**	**Beta (SE)**	**Sig.**	**R^2^**
BMI	0.590 (0.00)	0.001 *	0.455	WC	0.707 (0.00)	0.001 *	0.484	HC	0.427 (0.00)	0.004 *	0.392
TG	−0.002 (0.00)	0.983		TG	0.036 (0.00)	0.739		TG	0.045 (0.00)	0.706	
HDL-C	−0.033 (0.00)	0.779		HDL-C	0.041 (0.00)	0.734		HDL-C	−0.054 (0.00)	0.673	
ALT	0.123 (0.00)	0.294		ALT	0.036 (0.00)	0.763		ALT	0.226 (0.00)	0.055	
HbA1c	−0.108 (0.00)	0.261		HbA1c	−0.167 (0.00)	0.084		HbA1c	−0.090 (0.00)	0.372	

All data are presented as “R^2^” values of Multiple regression analysis. Significant values are shown as * *p* < 0.01.
